# Designs of InGaN Micro-LED Structure for Improving Quantum Efficiency at Low Current Density

**DOI:** 10.1186/s11671-021-03557-4

**Published:** 2021-06-03

**Authors:** Shiqiang Lu, Jinchai Li, Kai Huang, Guozhen Liu, Yinghui Zhou, Duanjun Cai, Rong Zhang, Junyong Kang

**Affiliations:** 1 Fujian Key Laboratory of Semiconductor Materials and Applications, CI Center for OSED, College of Physical Science and Technology, Xiamen University, Xiamen, 361005 People’s Republic of China; 2 Future Display Institute of Xiamen, Xiamen, 361005 People’s Republic of China

**Keywords:** InGaN, Micro-LED, Quantum efficiency, Low current density

## Abstract

Here we report a comprehensive numerical study for the operating behavior and physical mechanism of nitride micro-light-emitting-diode (micro-LED) at low current density. Analysis for the polarization effect shows that micro-LED suffers a severer quantum-confined Stark effect at low current density, which poses challenges for improving efficiency and realizing stable full-color emission. Carrier transport and matching are analyzed to determine the best operating conditions and optimize the structure design of micro-LED at low current density. It is shown that less quantum well number in the active region enhances carrier matching and radiative recombination rate, leading to higher quantum efficiency and output power. Effectiveness of the electron blocking layer (EBL) for micro-LED is discussed. By removing the EBL, the electron confinement and hole injection are found to be improved simultaneously, hence the emission of micro-LED is enhanced significantly at low current density. The recombination processes regarding Auger and Shockley–Read–Hall are investigated, and the sensitivity to defect is highlighted for micro-LED at low current density.

**Synopsis**: The polarization-induced QCSE, the carrier transport and matching, and recombination processes of InGaN micro-LEDs operating at low current density are numerically investigated. Based on the understanding of these device behaviors and mechanisms, specifically designed epitaxial structures including two QWs, highly doped or without EBL and p-GaN with high hole concentration for the efficient micro-LED emissive display are proposed. The sensitivity to defect density is also highlighted for micro-LED.

**Supplementary Information:**

The online version contains supplementary material available at 10.1186/s11671-021-03557-4.

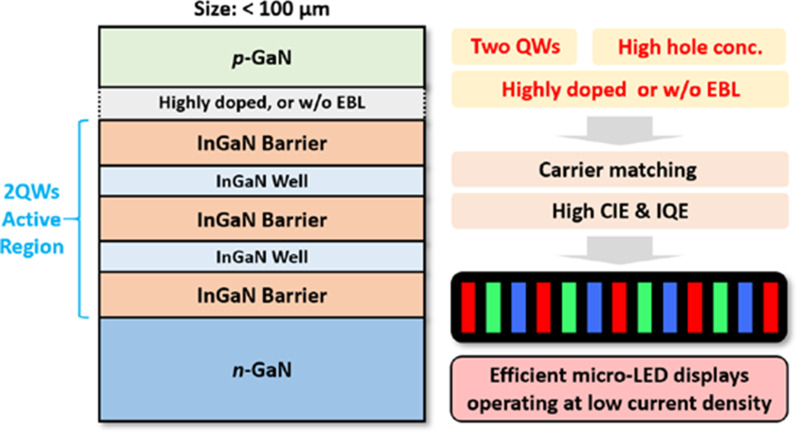

## Introduction

Group-III nitride based visible light-emitting diodes (LEDs) have a wide range of applications including signals, optical communication, information storage, backlights, displays, and general lighting (or solid-state lighting) [1, 2]. Since the first demonstration of InGaN micro-disk LED with a diameter of 12 μm by Jiang and his co-workers in 2000 [3, 4], the micro-LED has received expanding academic and industrial interests in the fields of high-resolution displays, visible light communication, bio-medicine, and sensing [5–8]. Compared with liquid–crystal display (LCD) and organic light-emitting diode (OLED), micro-LED has advantages of small size, high resolution, high luminous efficiency, high brightness, high color saturation, long operating lifetime, high response speed, and robustness, which make it the most promising candidate for the next generation display technology [9, 10]. Application scenarios of micro-LED display include high-end televisions (TV), laptops, handheld and mobile terminal devices, virtual reality (VR), augmented reality (AR), transparent display, and so on. According to Yole’s estimate, the market of micro-LED display will reach 330 million units by 2025 [11].

Traditional applications of group-III nitride LEDs, especially solid-state lighting, require the optical output power to be as high as possible [12]. In the last three decades, motivated by high-power applications, the modern research for nitride LEDs, including the design and optimization of the epitaxial structure, the study of the operating behavior and physical mechanism of the device, and the improvement of fabrication technology, is mainly focused on the large chip, high working current density, and high input/output power [12, 13]. Foremost, the active region of nitride LED has evolved from the simplest heterostructure and single quantum well (QW) at the early stage to today’s multiple quantum wells (MQWs) with 5 periods QWs, and the QW number even can reach 8 or 10 for several commercialized high-power devices [14–17]. The EBL was proposed to block the leakage of electrons at high injection current density, even it also can impede the hole injection at some certain level [18, 19]. For conventional high-power LEDs, the most substantial issue is the reduction of the external quantum efficiency (EQE) with the increased current density, which is known as efficiency droop. The intrinsic mechanism of this behavior is related to the indirect Auger recombination at high injected carrier density [20]. As for the fabrication, thin-film flip-chip and vertical injection geometry are developed to expand the power level of nitride LEDs [21, 22].

Considerable advances have been made for the traditional large-size high-power nitride LEDs, and some of the lessons learned can be leveraged for the study of micro-LEDs. However, micro-LEDs are still far different from their high-power counterparts. The different operating behaviors, mechanisms, and conditions of micro-LED can result in different challenges and research pathways [23, 24]. For traditional LEDs, the peak EQE is located at high current density, and the working current density is high and beyond the peak efficiency (> 30 A cm^−2^). But for the micro-LED emissive display, the working current density should be much lower and often in the range of 0.02 to 2 A cm^−2^ [24]. At this low current density, the EQE of traditional nitride LED is very low and not sufficient for the practical micro-LED display applications. By utilizing the benefits of V-pit to screen dislocations and enhance hole injection, Zhang and his co-works have created efficient InGaN-based LEDs with peak efficiency up to 24.0% at 0.8 A/cm^2^. However, the LED chips reported by Zhang et al. is still limited to of tranditional size (1 mm × 1 mm), which is much larger than that of micro-LED [25]. Moreover, many works have reported that the internal quantum efficiency (IQE) and EQE of micro-LEDs decrease as the chip size is reduced [26–28]. This size-dependent degradation is mainly attributed to the surface recombination and the sidewall damages induced by plasma-assisted dry etching. These side-wall effects contribute to the Shockley–Read–Hall (SRH) non-radiative recombination, then decrease the quantum efficiency, and become much more serious with a smaller chip size because of the larger specific surface/side-wall area compared with the active region of the device [29–31]. To address this issue, sidewall passivation using dielectric materials and wet etching using buffered hydrofluoric acid or photoelectrochemical method were proposed to minimize these effects to a certain level for the micro-LED [31–33]. However, even improved by the sidewall passivation, the peak EQE of micro-LEDs (with a size smaller than 60 μm) is still lower than 25%, and dramatically decreased to several percent at a current density lower than 2 A cm^−2^ [34, 35]. Especially for the InGaN-based red micro-LED, the currently reported EQE is quite limited to lower than 1%, due to the strong polarization and poor crystal quality [36]. Moreover, with the chip size reduced, the position of peak EQE also moves to a higher current density, which will further impede high efficiency at low current density [26].

Therefore, improving quantum efficiency at low current density becomes the large challenge and critical scientific issue for the micro-LED emissive display application. For this purpose, peak efficiency should be increased, and the onset position of efficiency should be shifted to an appropriate lower current density. Previously works mainly focused on the improvement of fabrication technology such as sidewall passivation. To improve efficiency, investigating the operating behaviors and physical mechanisms of micro-LED at low current density, which is still relatively unexplored and lack understanding, is also essential. In addition, to create device that can improve the efficiency at low current density with a maximum value, the epitaxial structure of micro-LED also needs to be re-designed and optimized, which should be quite different from their traditional large-size high-input/output counterparts. For now, the specifically designed epitaxial structure for the micro-LED emissive display operating at low current density remains lacking.

In this work, the unique challenges of micro-LED for the display application operating at low current density are highlighted, and potential solutions to address them are proposed. Using the software Advanced Physical Model of Semiconductor Devices [37], we numerically investigate the operating behaviors and physical mechanisms of InGaN micro-LEDs at various current density from 200 to 0.1 A/cm^2^. The band diagram, wavefunction, and polarization field are simulated and analyzed for the QCSE of micro-LED, and a severer QCSE at low current density is confirmed. Influences of QW number, *p*-type doping concentration and AlGaN EBL on the carrier transport, carrier matching, radiative recombination and quantum efficiency of micro-LED are investigated systemically. The effect and mechanism regarding the SRH and Auger recombination are also discussed. Based on the simulation and analysis, an optimized epitaxial structure specifically designed for micro-LED operating at low current density is proposed.

## Device Structures and Simulation Methods

**Fig. 1:**
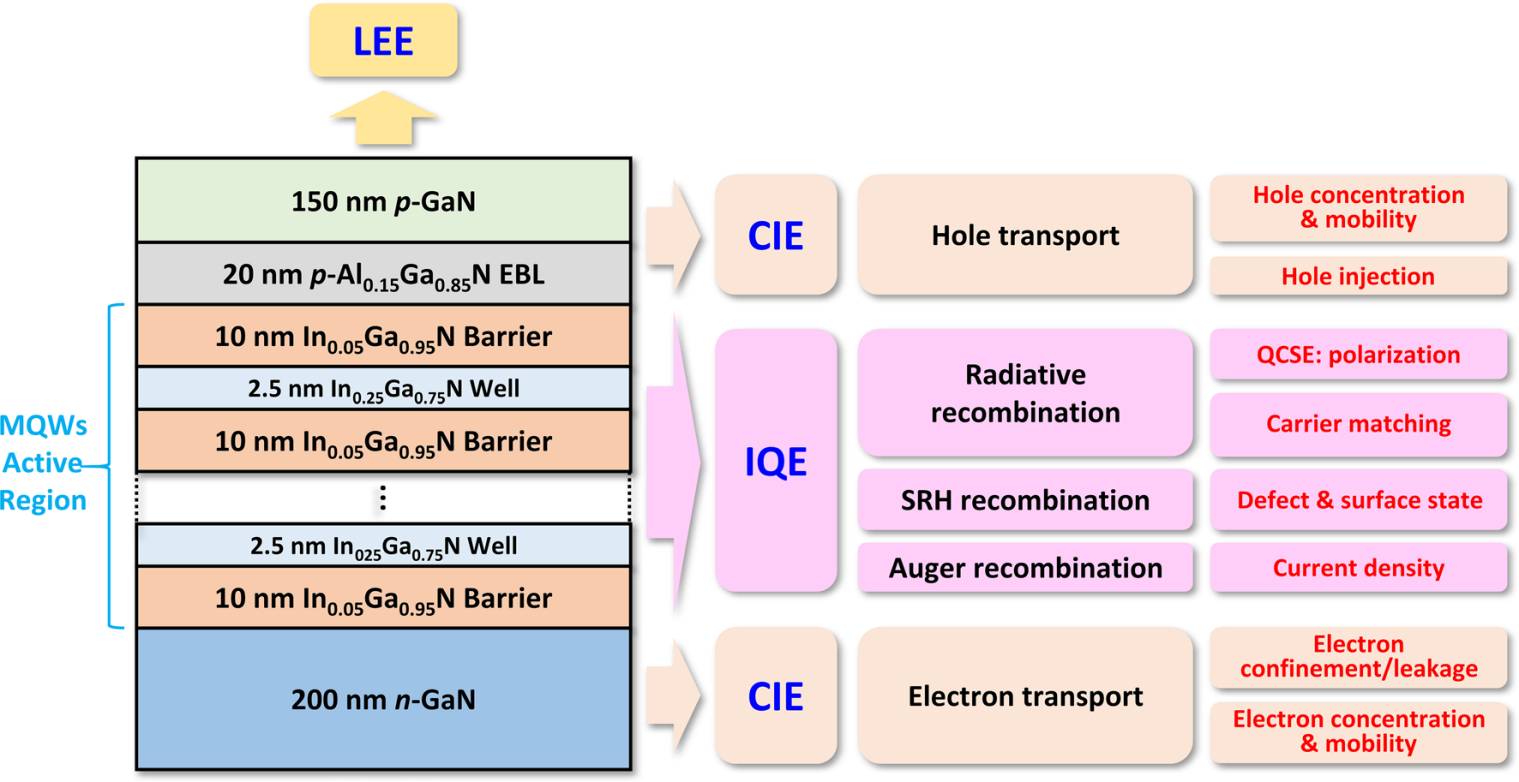
Schematic illustration of the InGaN/GaN-based blue light-emitting-diode used for the simulation and the analysis of efficiency for LED

In this study, the common structure of blue InGaN micro-LED with a rectangular chip size of 60 × 60 μm and a peak emission wavelength around 465 nm is used for the simulation. Figure 1 shows that the blue micro-LED is composed of 200 nm *n*-GaN layer, MQWs active region, 20 nm *p*-Al_0.15_Ga_0.85_ N EBL and 150 nm *p*-GaN layer. The MQWs active region consists of 8, 5, 3, 2 or 1 periods with 2.5-nm-thick In_0.25_Ga_0.75_ N QW embedded in 10-nm-thick In_0.05_Ga_0.95_ N quantum barrier (QB). The In composition of MQWs is adjusted and optimized to achieve the desired blue emission wavelength. The doping concentration of *n*-GaN, *p*-AlGaN EBL, and *p*-GaN are 2 × 10^18^ cm^−3^, 3 × 10^18^ cm^−3^, and 1 × 10^19^ cm^−3^, respectively.

The optical and electrical properties of micro-LEDs are numerically investigated using the software Advanced Physical Model of Semiconductor Devices [37]. In the simulation, 50% of the interface charge densities caused by spontaneous and piezoelectric polarization is assumed [38]. Except for the specifically mentioned, the SRH lifetime and Auger recombination coefficient are set as 100 ns and 1 × 10^–31^ cm^6^s^−1^, respectively [39, 40]. The band offset ratio is assumed 0.7/0.3 [41], the light extraction efficiency is fixed at 70%, and the operating temperature is 300 K. Other material parameters used in the simulation can be found in Ref [42].

## Results and Discussions

The Wall-plug efficiency (WPE) of LED can be expressed as follows:

1 $${\text{WPE}} = \frac{{P_{{{\text{out}}}} }}{{I_{{{\text{op}}}} \times V}} = \eta_{{{\text{EQE}}}} \frac{\hbar \omega }{{e \times V}} = \eta_{{{\text{EQE}}}} \times \eta_{{{\text{elect}}}} ,$$

where *P*_out_ denotes the optical output power, *I*_op_ is the operating current, *V* is the drive voltage, ℏ*ω* is the photon energy, and *η*_elect_ is the electrical efficiency. EQE *η*_EQE_ is a product of current injection efficiency (CIE) *η*_CIE_, IQE *η*_IQE_ and light extraction efficiency (LEE) *η*_*LEE*_, as descrebed by the following equation:

2 $$\eta_{{{\text{EQE}}}} = \eta_{{{\text{CIE}}}} \times \eta_{{{\text{IQE}}}} \times \eta_{{{\text{LEE}}}} .$$

Furthermore, according to the ABC model [43], *η*_IQE_ can be expressed as follows:

3 $$\eta_{{{\text{IQE}}}} = \frac{{R_{{{\text{rad}}}} }}{{R_{{{\text{rad}}}} + R_{{{\text{SRH}}}} + R_{{{\text{Auger}}}} }},$$

where *R*_rad_ is the radiative recombination rate, *R*_SRH_ is the SRH non-radiative recombination rate, and *R*_Auger_ is the Auger recombination rate. Figure 1 shows the different kinds of efficiency and the related physical mechanisms and factors.

*R*_rad_ and CIE must be maximized, electrical efficiency and LEE must be improved, and SRH and Auger recombination must be reduced to improve the overall efficiency of micro-LED operating at low current density. System-level approaches are required to address all these challenges. Except for light extraction, these challenges will be discussed in the following sections, and potential solutions for creating efficient epitaxial structure of micro-LED will also be proposed.

### QCSE at Low Current Density: Internal Polarization Field

Polarization-induced QCSE is one of the dominant factors that limit the IQE of nitride LED [44]. QCSE has been widely studied for the traditional large-size high-power LED, but still lacks sufficient discussion in the context of micro-LED specific applications. Therefore, this important effect is first investigated. The active region of micro-LED discussed here is constructed by 5 period QWs, which is the most commonly used QW number for traditioanl nitride LED.

**Fig. 2:**
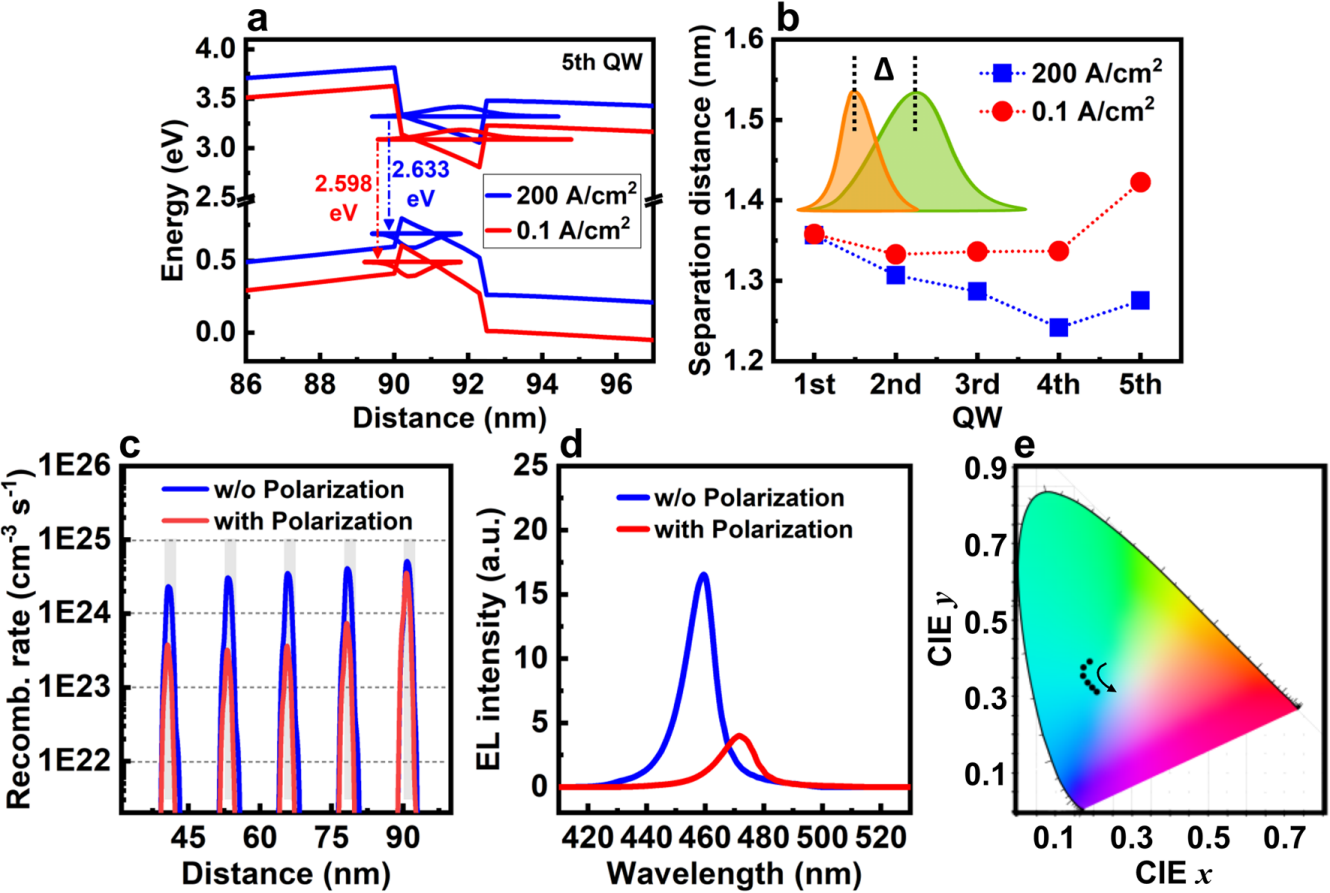
**a** Energy band diagrams and the related first-level electron and hole wavefunctions of the fifth QW at 200 and 0.1 A/cm^2^, respectively. **b** Separation distance of the peak position of electron and hole wavefunctions at 200 and 0.1 A/cm^2^, respectively. **c** Radiative recombination rates and **d** EL spectra calculated at 0.1 A/cm^2^ without and with polarization, respectively. **e** Color points created from the blue, green, and red LEDs with current density from 0.1 to 20 A/cm^2^ plotted on the 1931-CIE (x, y) chromaticity diagram

Figure 2a shows the energy band diagrams and the related first-level electron and hole wavefunctions of the fifth QW at 200 and 0.1 A/cm^2^. QB and QW experience a large band bending, leading to the spatial separation of electron and hole wavefunctions. Moreover, band bending is stronger at low current density, indicating a stronger QCSE. This phenomenon is attributed to the weak screening effect with less nonequilibrium carriers at low injection current density (see Additional file 1: Fig. S1a-d, and related discussion) [41]. Figure 2b shows that a severer QCSE enhances the spatial separation of carrier wavefunctions at low current density, which leads to a lower radiative recombination rate. The radiative recombination rates and EL spectra without and with polarization effect, i.e., without and with QCSE, are further calculated to show how the electron–hole separation quantitatively reduces emission at low current density. Figure 2c, d shows that the integral intensity of radiative recombination rate and EL intensity are reduced approximately 84.0% and 72.3% by QCSE, respectively. These results indicate that it is more difficult to improve the efficiency for micro-LED than their traditional high-power cousions due to the enhanced QCSE at low current density.

Moreover, the accurate and stable control of emission wavelength at different injection current densities is critical for micro-LED display, because it determines various important parameters, such as brightness, color accuracy, and saturation. However, a severer QCSE at low current density makes this a large challenge, especially for green and red micro-LEDs that require a higher Indium composition. As a direct result of the increased band bending in InGaN QWs with a higher Indium composition and a stronger QCSE, the wavelength shift versus current density becomes more pronounced due to the screening effect. The EL spectra of InGaN-based red, green, and blue micro-LED with various current densities are further calculated to show how the spectra shift affects color points in the display. Additional file 1: Figure S2 shows that from 0.1 to 20 A/cm^2^, the peak wavelengths blue shift by approximately 15.8, 6.6, and 1.7 nm for red, green, and blue micro-LED, respectively. The color points created by combining the red, green, and blue micro-LEDs are also calculated, as summarized in Additional file 1: Table S1. Figure 2e illustrates the corresponding 1931-CIE (x, y) chromaticity diagram. Clearly, the color of the emission from RGB micro-LEDs greatly changed from green to blue-green when current density increases. The 1931-CIE x value crosses from 0.1676 to 0.2084, and the 1931-CIE y value crosses from 0.3891 to 0.3106. This substantial change of color point versus current density greatly deteriorates the performance of micro-LED display.

Many reports have suggested several approaches to suppress the QCSE, such as using InGaN/AlGaN ultra-thin superlattice barriers [45], InGaN/GaN/AlGaN/GaN MQWs with GaN interlayer barrier [46], wrinkled MQWs [47], and inserting a strain-relief layer before MQWs [48]. But QCSE is induced by the intrinsic properties of *c*-plane nitride material. Several adjustments of MQWs are not sufficient to suppress this effect completely. Recently, a decent efficiency has been achieved for non-polar nitride LED. If the cost of non-polar GaN substrate can be reduced, non-polar LED can be a ideal solution for decreasing the QCSE and realizing stable full-color emision [49].

### Carrier Transport and Matching

The electron (*J*_*n*_) and hole (*J*_*p*_) current density can be expressed as follows:

4 $$J_{n} = \sigma_{n} \left| {\varvec{E}} \right| = nq\mu_{n} \left| {\varvec{E}} \right|,$$

5 $$J_{p} = \sigma_{p} \left| {\varvec{E}} \right| = pq\mu_{p} \left| {\varvec{E}} \right|,$$

where *σ*_*n*_ and *σ*_*p*_ denote the conductivity, *n* and *p* are the concentration, *μ*_*n*_ and *μ*_*p*_ are the mobility for the electron and hole, respectively, and ***E*** denotes the electric field. In nitride, hole concentration is an order of magnitude lower than the electrocn [50], and hole mobility is two orders of magnitude lower than electron [51]. This asymmetry of concentration and mobility leads to the mis-matching of carrier flux (*J*_*n*_ > *J*_*p*_) and deteriorates the performance of LED in following two ways.

*Current injection efficiency*: Current injection efficiency *η*_CIE_ represents the ratio of the recombination current *J*_recomb_ to the total current *J*_total_, as following equation [52]:

6 $$\eta_{{{\text{CIE}}}} = \frac{{J_{{{\text{recomb}}}} }}{{J_{{{\text{total}}}} }} = \frac{{J_{{n{\text{ - recomb}}}} + J_{{p{\text{ - recomb}}}} }}{{J_{n} + J_{p} }} = \frac{{2 \times J_{p} }}{{J_{n} + J_{p} }}.$$

*J*_recomb_ depends on the smaller one of carrier currents, i.e., hole current. Equation (6) confirms that the carrier mis-matching (*J*_*n*_ > *J*_*p*_) limits the maximization of CIE.

*Radiative recombination rate*: The radiative recombination rate *R*_rad_ is described by Fermi’s golden rule as the following equation:

7 $$R_{{{\text{rad}}}} = C \times \smallint {\text{d}}E_{cv} hv_{cv} \left| {\overline{{M_{T} }} \left( {E_{cv} } \right)} \right|^{2} \rho_{r} \left( {E_{cv} } \right)f_{v} \left( {1 - f_{c} } \right),$$

where *C* is a constant, *E*_*cv*_ is the transition energy, *h* is the Planck constant, *ν*_*cv*_ is the frequency of the generated light, *ρ*_*r*_ is the reduced density of states, *f*_*c*_ and *f*_*v*_ are the Fermi–Dirac distributions, and *|M*_*T*_*|*^2^ is the momentum matrix element [53]. Less hole and additional electron accumulation in the QW can lead to the expansion of crystal lattice and the buildup of tensile strain. Under this stress variation, the charge densities of quantum levels around the valence band maximum are reduced. This further decresases the optical transition probability and reduces *R*_rad_ according to Eq. (7). In this manner, the local carrier mis-matching in a single QW also limits the IQE. More specific discussion about this topic can be found in previous reports [54–56].

In the following sections, the influences of QW number, *p*-type doping concentration, and EBL structure on the carrier transport will be analyzed to deterimine the best carrier matching conditions. Finally, an optimized epitaxial structure for the efficient micro-LED display operating at low current density will be proposed.

#### Carrier Mis-Matching in 5QWs

First, the carrier transport properties of blue micro-LED with 5QWs are simulated. The distributions of carrier concentration at 200 A/cm^2^ and 0.1 A/cm^2^ are illustrated in Additional file 1: Fig. S3a and b, respectively. The inhomogeneous distribution in 5 QWs can be observed both at high and low current densities. Additional file 1: Figure S3c and d shows that the electron mobility in MQWs (684 cm^2^V^−1^ s^−1^) is two orders of magnitude higher than the hole mobility (10 cm^2^V^−1^ s^−1^). Hence, electrons may just inject into, then pass through MQWs without participating in the recombination, leading to the electron leakage problem and a low CIE [51].

**Fig. 3:**
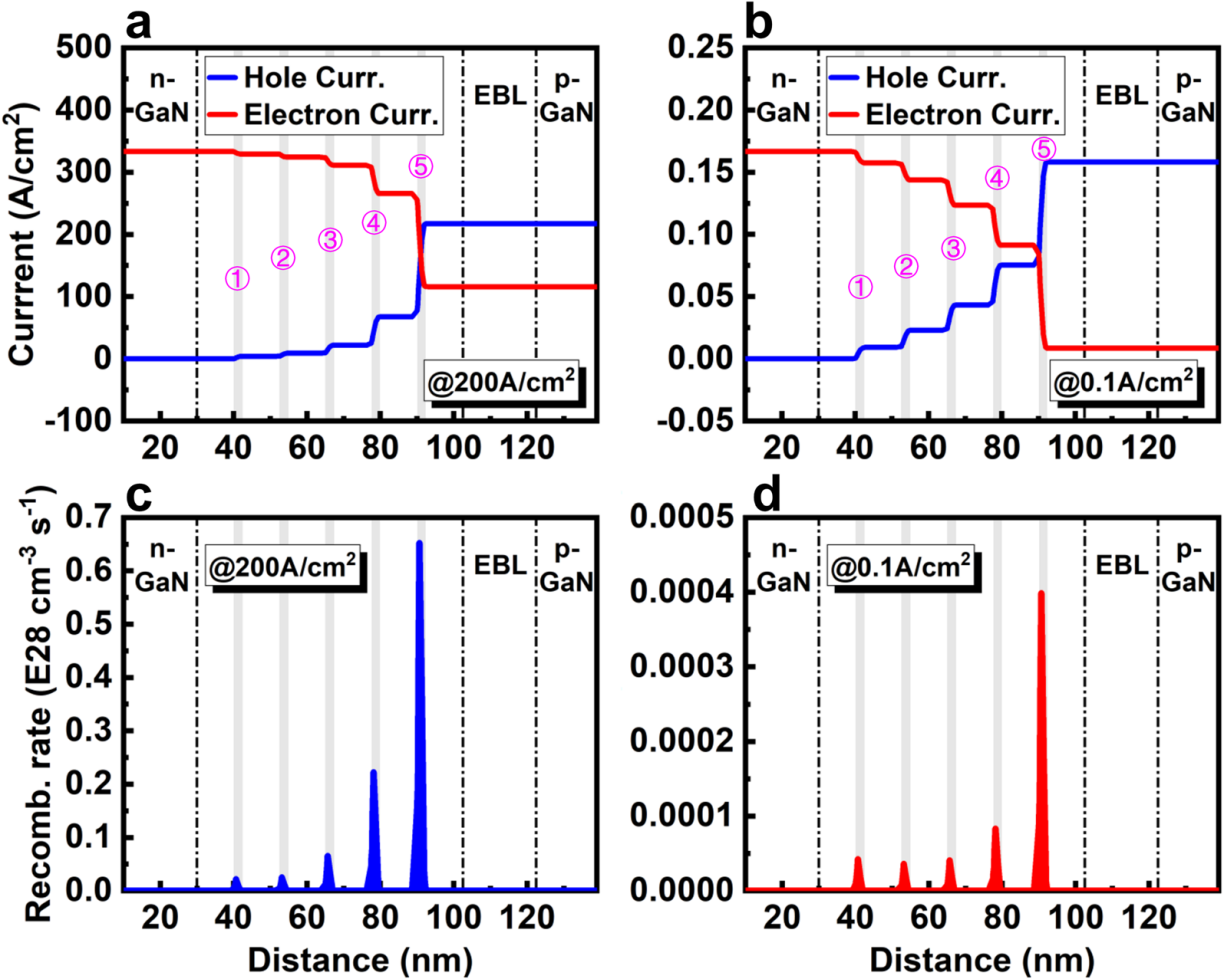
Carrier current distribution of LED with 5QWs **a** at 200 A/cm^2^ and **b** at 0.1 A/cm^2^. Radiative recombination rates of LED with 5QWs **c** at 200 A/cm^2^ and **d** at 0.1 A/cm^2^

Figure 3a shows the distribution of electron and hole current density at 200 A/cm^2^. Total hole current density (217.4 A/cm^2^) is only 65.2% of the electron (333.3 A/cm^2^), indicating a severe mis-matching of carrier and a low CIE. The leakage electron current is as high as 116.0 A/cm^2^, which deteriorates both the radiative efficiency and the hole injection. As shown in Fig. 3b, the leakage electron current is only 0.01 A/cm^2^, and the calculated *η*_*CIE*_ is as high as 95% at 0.1 A/cm^2^. These results indicate that achieving a high CIE is easier at low current density. However, except the 5th QW where *J*_*p*_ can be equal to *J*_*n*_, the carrier mis-matching and additional electron accumulation are quite severe in other four QWs (QW 1, 2, 3 and 4) both at high and low current density. At 200 A/cm^2^, the electron current densities of these four QWs are 120, 43, 16 and 5 times higher than the hole current density (Fig. 3a). At 0.1 A/cm^2^, they are 23, 9, 4, and 2 times higher than the hole current (Fig. 3b). Based on Eq. (7), this great carrier mis-matching evidently decreases the radiative recombination rate of these four QWs. Therefore, radiative recombination rates in these four QWs are just about 3.4%, 4.0%, 10.1%, and 34.2% at 200 A/cm^2^, and 11.3%, 10.1%, 10.7% and 21.2% at 0.1 A/cm^2^ compared with the 5th QW. These carrier mis-matching and low radiative emission finally reduce the monolithic efficiency of micro-LED.

#### Factors Influencing Carrier Transport and Matching

*QW number*: For traditional large-size LED operating at high current density, MQWs with 5, even 8 or 10 periods must be used to achieve a high optical output power. However, for the micro-LED emissive display, the output power is much smaller and the working current density is much lower. As discussed in the previous section, even at low current density, the carrier mis-matching remains quite severe in 5QWs, and only one QW can achieve the best matching condition. From this perspective, for the micro-LED operating at low current density, the active region with less QW number should be a better design for improving the efficiency due to the improved carrier matching.

**Fig. 4:**
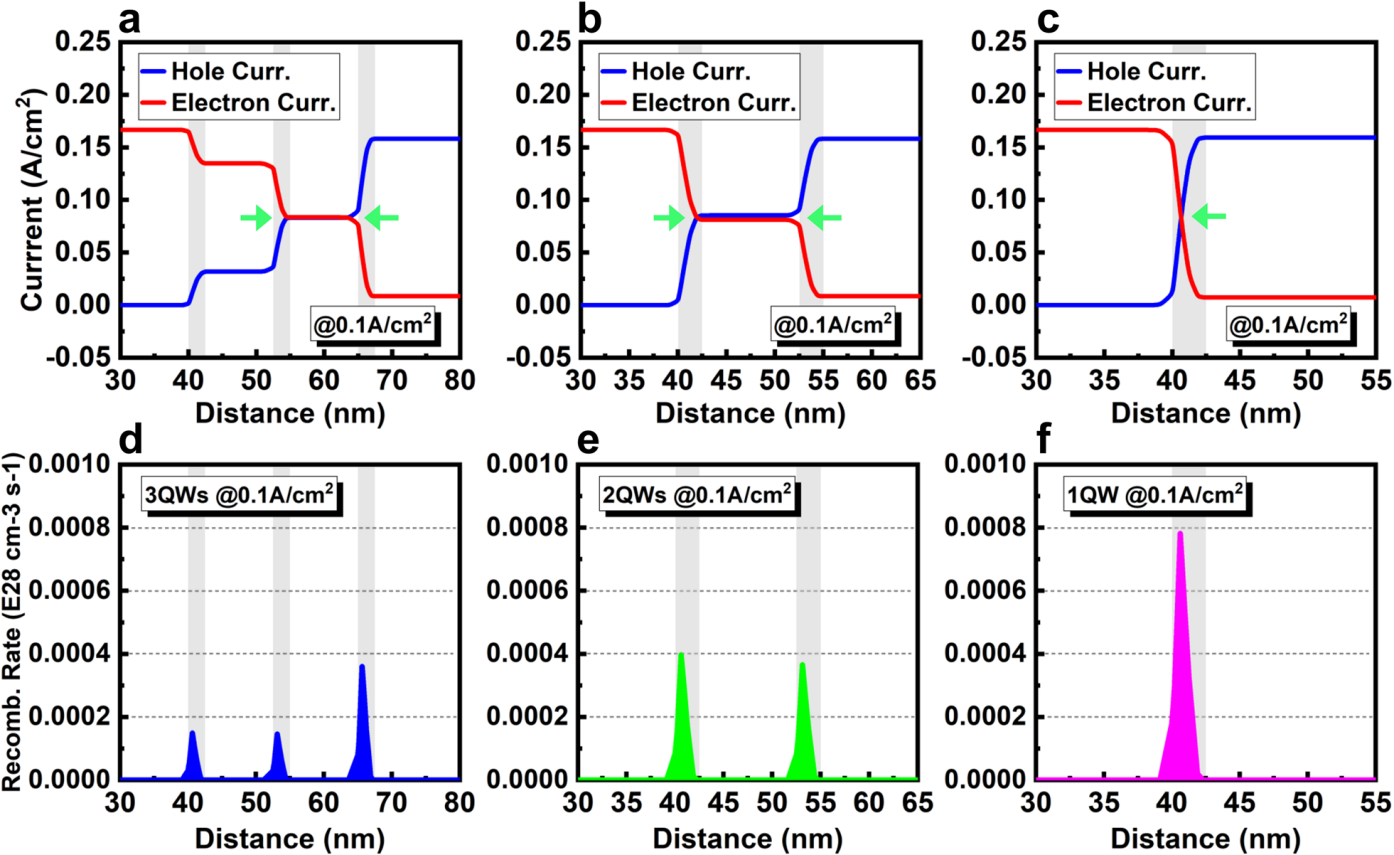
Carrier current distribution of LED with **a** 3QWs, **b** 2QWs, and **c** 1QW at 0.1 A/cm^2^. Radiative recombination rates of LED with **d** 3QWs, **e** 2QWs, and **f** 1QW at 0.1 A/cm^2^

The effect of the QW number on the micro-LED is investigated to verify our assumption. Figure 4a–f shows the carrier current density and radiative recombination rate at 0.1 A/cm^2^ of LEDs with 3QWs, 2QWs, and 1QW, respectively. The current curves have only one intersection point (one point of best carrier matching, *J*_*n*_ = *J*_*p*_) due to the monotonically decreasing tendency of current, but with fewer QWs, as the cases of 3QWs and 2QWs, two intersection points can be achieved in two different QWs (Fig. 4a, b). In other word, the carrier mis-matching in MQWs can be partially overcome with fewer QWs. Especially for the 2QWs, with appropriate adjustment, the perfect matching of carrier flux can be achieved in all two QWs. Radiative recombination rate is also higher in the 2QWs than 3QWs and 5QWs because the carrier consumption by the radiative recombination is more concentrated in the active region with fewer QWs (Figs. 3d, 4d, e). Undoubtedly, the best carrier matching is in the LED with only one QW, and the radiative recombination rate is also highest for the 1QW, as shown in Fig. 4c, f.

**Fig. 5:**
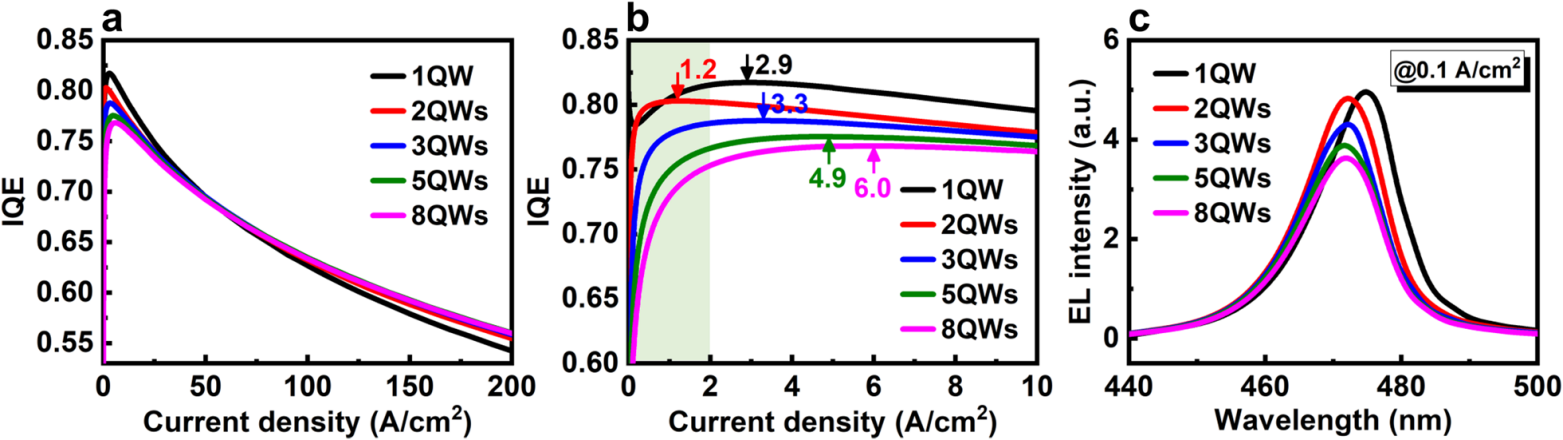
**a** IQE curves and **b** IQE curves at low current density of LED with 8, 5, 3, 2, and 1 QWs. **c** EL spectra of LED with 8, 5, 3, 2, and 1 QWs at 0.1 A/cm^2^

Figure 5a, b shows IQE as a function of the current density between 0 to 200 A/cm^2^ and 0 to 10 A/cm^2^, respectively. For a current density higher than 50 A/cm^2^, IQE decreases when the QW number is reduced. By contrast, IQE with a current density lower than about 30 A/cm^2^ increases when QW number decreases. At 0.1 A/cm^2^, IQE values for 8, 5, 3, 2, and 1 QWs are 55%, 62%, 69%, 77%, and 78%, respectively. Moreover, as shown in Fig. 5b, the position of peak IQE also moves from 6.0 A/cm^2^ in 8 QWs to the lowest current density approximately 1.2 A/cm^2^ in 2QWs. The IQE curves at low current density (< 1 A/cm^2^) also become steeper and sharper with a lower QW number, indicating that achieving the highest efficiency is easier and quicker. This is quite beneficial for improving the efficiency at low current density. The physical mechanism behind this tendency of IQE can be explained by the better matching of carrier flux, and more concentrated, stronger radiative emission in the active region with fewer QW number. As shown in Fig. 5c, compared with 8 QWs, the integral EL intensity of 5, 3, 2, and 1 QWs at 0.1 A/cm^2^ are increased about 6.1%, 14.8%, 28.4%, and 32.1%, respectively. This result confirms that not only the efficiency but also the output power is improved with fewer QW number.

The simulation results show that 1QW has the highest EL intenisty and maybe the best structure for micro-LED operating at low current density. However, experimentally, it is difficult to epitaxially grow just one QW which has flat surface and sharp interface. Moreover, for the 1QW structure, the position of peak IQE increases slightly to 2.9 A/cm^2^, and the shape of IQE curve also slightly changed. This can be explained by the special circumstance of the single QW. Compared with other QWs, the QW adjoined to the EBL has a special polarization environment and it is considered as a “problem QW.” This topic will be discussed with more details in the section of *AlGaN EBL*. Considering these reasons, the 2QWs should be the best active region design, which has the similar good matching of carrier flux, close IQE value, and EL intensity to 1QW. Therefore, in the following sections, all simulations are based on the micro-LED with 2QWs.

**Fig. 6:**
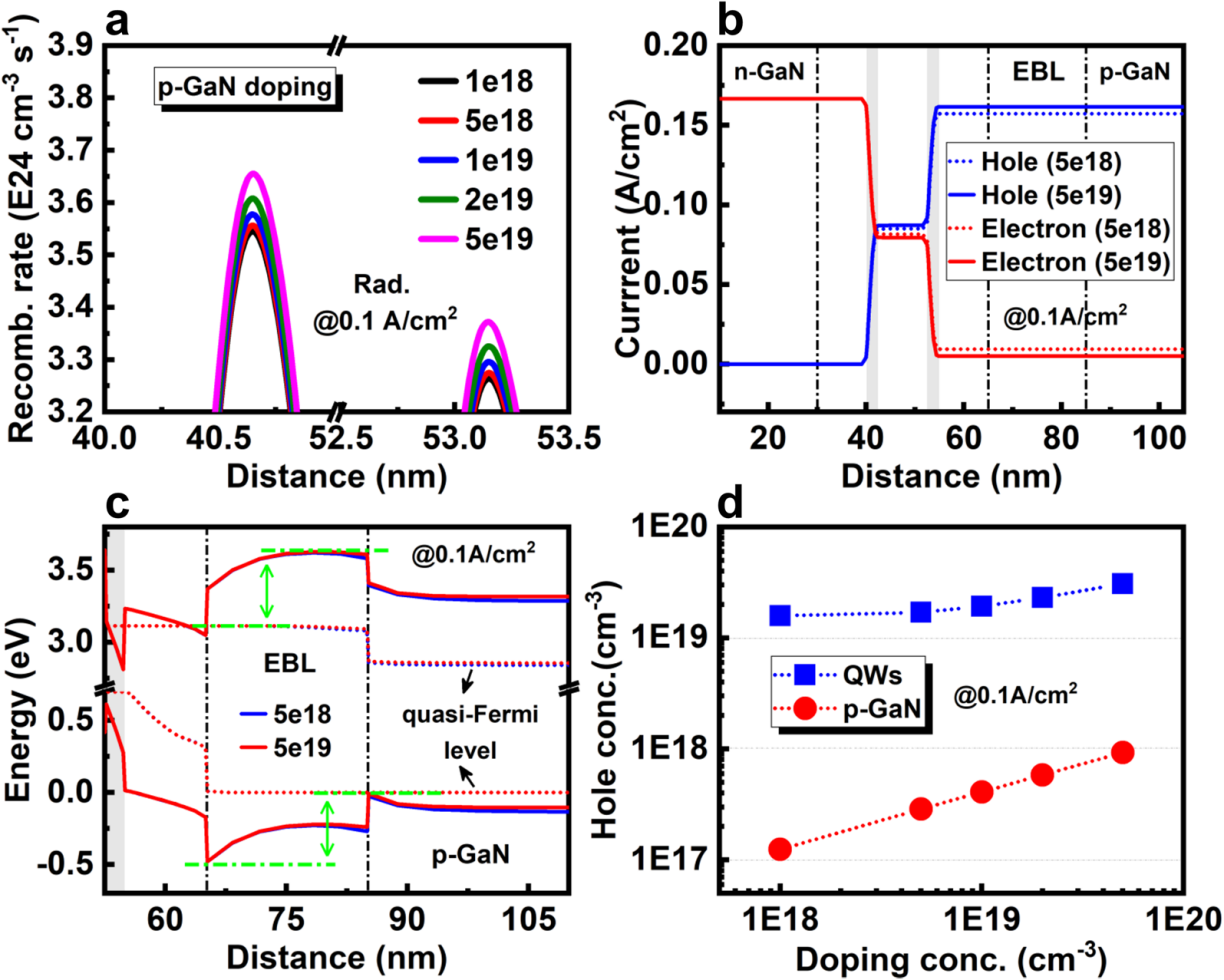
**a** Radiative recombination rates of 2QWs LED with various doping concentrations in p-GaN. **b** Carrier current distribution with different doping concentrations in *p*-GaN. **c** Enlarged energy band diagrams of EBL with different doping concentrations in *p*-GaN. **d** Average hole concentration in *p*-GaN and QWs with various doping concentrations of *p*-GaN

*P-GaN doping concentration*: The performance of 2QWs LEDs with different *p*-type doping concentrations in *p*-GaN is further investigated. As shown in Fig. 6a, when the doping concentration of *p*-GaN increasing from 1 × 10^18^ cm^−3^ to 5 × 10^19^ cm^−3^, the radiative recombination rates at 0.1 A/cm^2^ further increases about 3.1% and 3.0% for the two QWs. Figure 6b shows that the total hole current density increases from 0.157 to 0.162 A/cm^2^, meanwhile, leakage electron current density is reduced from 0.009 to 0.005 A/cm^2^ with the increase of doping concentration. It's worth noting that the recombination current in the QW near *n*-side is higher than the QW near *p*-side (Fig. 6b). Therefore, the radiative recombination rate near the *n*-side QW is also slightly higher than that near the *p*-side QW. As shown in Fig. 6c, one can find that the energy barrier for carriers in EBL, which is defined as the energy distance between the electron/hole quasi-Fermi level and the highest conduction band or lowest valence band, are almost unchanged under differnt doping concentration of *p*-GaN, that is, the hole injection is not improved by increasing doping concentration. Figure 6d shows the average hole concentration in the *p*-GaN and QWs. The hole concentration in the *p*-GaN is almost exponentially dependent on the doping concentration. Inside the QWs, the hole concentration is increased approximately twice from 1.59 × 10^19^ cm^−3^ to 3.08 × 10^19^ cm^−3^ with a higher doping concentration. These results indicate that the increased hole concentration is the main contribution for the improvement of radiative recombination. Therefore, even at low current density, the *p*-type doping problem of nitride remains notable, and enhancing the doping efficiency and hole concentration is still beneficial for the efficiency of micro-LED.

*AlGaN EBL*: In the last decades, a 10–20 nm *p*-type AlGaN EBL has become a standard structure for traditional nitride-based LEDs. This EBL is considered to block the electron leakage and suppress the efficiency droop under high injected current density. In spite of this, it is worth noting that the EBL is still a very complicated, subtle structure for the LED. It involves many important factors, including Al composition, *p*-type doping efficiency of AlGaN, band offset ratios, and polarization effect. Each of them can influence the band structure and carrier transport substantially, then determines the effectiveness of EBL. For the construction of EBL, thickness, composition, and doping concentration must be considered and optimized carefully to balance the enhancement of electron confinement and the blocking of hole injection, otherwise, the opposite may happen, and the performance of LED might deteriorate. For micro-LED, the effectiveness of EBL for operating at low current density must be reconsidered, which may be different with the case of traditional high input/output LED.

**Fig. 7:**
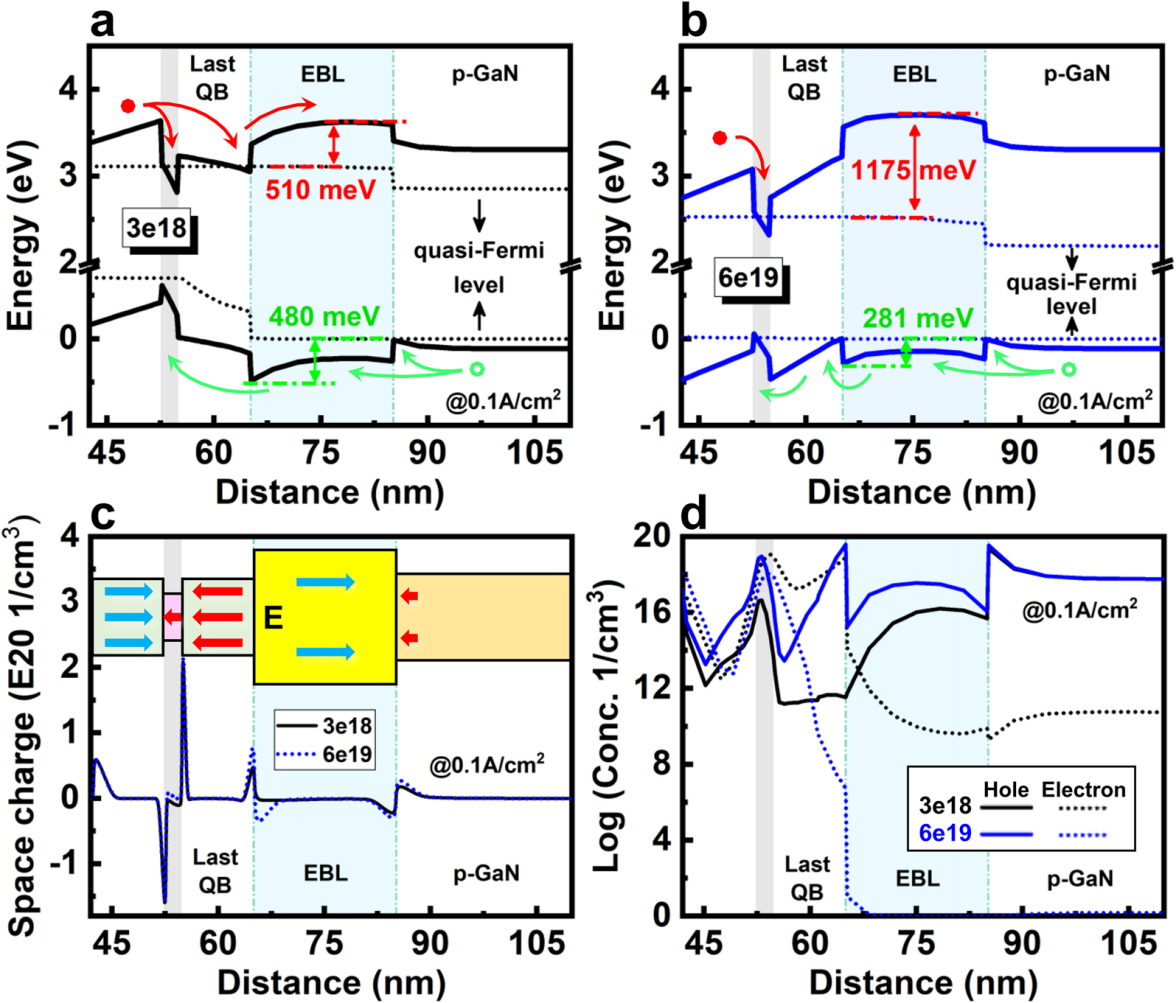
Energy band diagrams of 2QWs LED with **a** 3 × 10^18^ cm^−3^ and **b** 6 × 10^19^ cm^−3^ doping concentration in AlGaN EBL. **c** Space charge density and **d** carrier concentration distribution of 2QWs LED with 3 × 10^18^ and 6 × 10^19^ cm^−3^ doping concentration of EBL. The inset of **c** shows the direction of polarization fields

*a. Doping concentration of EBL*: First, the effect of EBL doping concentration on carrier transport at low current density is investigated. The thickness and Al composition of EBL are fixed as 20 nm and 0.15, respectively. Considering the low solubility of Mg dopant in AlGaN, the crystal degradation, and compensation effect by over-doping [50], the doping concentration of EBL is first set as be 3 × 10^18^ cm^−3^. Figure 7a shows the corresponding energy band structure. Clearly, despite the existence of EBL, the electron leakage out of the QW still can be caused by the insufficient electron confinement due to the downward bending of the last QW and EBL. A new energy valley under the electron quasi-Fermi level appears at the interface of last QB and EBL. Thus, electrons would escape from QW and accumulate in this area. This distortion of band structure makes the EBL relatively ineffective, and it can be contributed to the polarization effect. As shown in Fig. 7c, the strong polarization induces a large amount of charges at the interfaces. Due to the unbalanced polarization charges are positive at the interface of the last QB/EBL, a large electrostatic field pointing from the *p*-side to the *n*-side builds up in the last QB, which is opposite to the fields in other QBs and EBL. These electrostatic fields pull down the energy band of the last QB and EBL. Moreover, the electric fields in the last QW and last QB both can attract electrons and drive them out of the active region into the *p*-layer. This can be observed in the carrier concentration diagram, as shown in Fig. 7d. The dotted black line indicates that a part of the electrons escape from the active region and accumulate at the interface of last QB/EBL. In the EBL and *p*-GaN, the leakage electron remains relatively high.

On the other hand, the EBL also introduces a potential barrier as high as 480 meV for hole injection. Moreover, as shown in Fig. 7a, c, an energy valley close to the hole quasi-Fermi level appears at the interface region between the EBL/*p*-GaN due to the polarization effect. As indicated by the solid black line of Fig. 7d, most of the holes are blocked by the EBL and thus, accumulate at the energy valley of the EBL/*p*-GaN interface. Owing to this inefficient carrier transport, the last QW is considered as a “problem QW,” and the EBL only has a low capability for electron confinement, and should be responsible for the poor hole injection. Compared with the traditional high input/output LED, this polarization induced ineffectiveness of the EBL function could be particularly severe for the micro-LED due to the enhanced polarization effect by less carrier screening at low current density.

Band engineering by increasing the doping concentration of EBL is a possible method to improve the electron confinement and hole injection. The activation energy of Mg dopant in AlGaN EBL is higher than GaN, therefore, even under a similar doping concentration, the active hole concentration in EBL remains much lower than *p*-GaN. The lower hole concentration could further separate the hole quasi-Fermi level and valence band, then increase barrier height. Based on this analysis, the doping concentration of EBL needs to be much higher than *p*-GaN. Considering the doping limitation in actual experiment, 6 × 10^19^ cm^−3^ is selected as a new doping concentration in the EBL. As shown in Fig. 7b, by increasing the doping concentration, the valence band of EBL is lifted due to the alignment of hole quasi-Fermi level, resulting in a reduced hole energy barrier of 281 meV. Moreover, the high *p*-type doping also helps lower the electron quasi-Fermi level with respect to the conduction band in EBL, hence increasing the effect barrier for electron leakage to 1175 meV. These changes improve electron confinement and hole injection. Figure 7d shows that compared with the doping concentration of 3 × 10^18^ cm^−3^, the hole concentration in the active region is greatly increased, and the leakage electron in the EBL and *p*-GaN is reduced to almost zero. However, the energy valley at the interface between the EBL/*p*-GaN still exists. Moreover, the upward of valence band also introduces a new energy valley for the hole accumulation at the interface between the last QB/EBL, which can be confirmed by the hole concentration distribution in Fig. 7d. These energy valleys can impede the hole injection into QWs, hence compensating the advantage of high doping concentration.

**Fig. 8:**
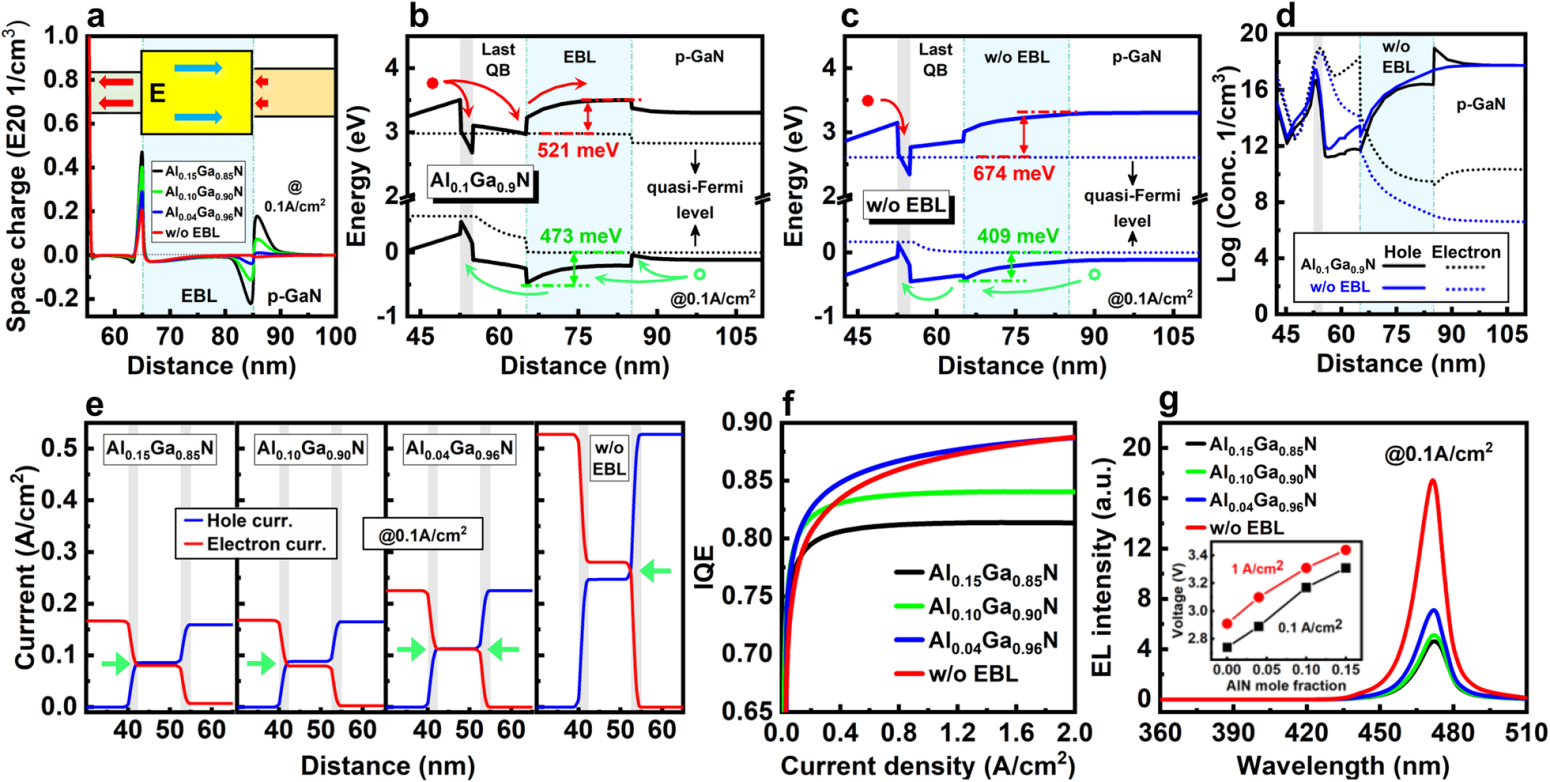
**a** Space charge density distribution of 2QWs LED with different EBL. The inset shows the direction of polarization field. Energy band diagrams of 2QWs LED **b** with Al_0.1_Ga_0.9_ N EBL and **c** without EBL. **d** Carrier concentration distribution, **e** carrier current density, **f** IQE and **g** EL spectra of 2QWs LED with different EBL. The inset of **g** shows the working voltages at 0.1 and 1 A/cm^2^ with different EBLs

*b. Al composition of EBL*: Compared with the increase of doping concentration, reducing the composition of AlGaN EBL may be an easier, more efficient method to improve the carrier transport at low current density. The effectiveness of EBL is sensitively dependent on Al composition, band offset, and polarization effect. Increasing the Al composition of EBL can increase the band offset between the last QB/EBL, which increases the electron barrier height. However, as shown in Fig. 8a, the polarization-induced charges at the interfaces also increase accordingly, which pull down the electron barrier height. Two mechanisms have the opposite effect for confining electrons.

Based on this analysis, the carrier transport of 2QWs micro-LEDs with different EBL structures at 200 and 0.1 A/cm^2^ are investigated. The result of effective energy barrier heights of different EBL are summarized in Table 1. First of all, both the electron and hole energy barriers at low current density are much higher than the cases of at high current density due to the lower nonequilibrium carrier population. A higher energy barrier can improve the electron confinement, but also severely impedes the hole injection at low current density. This indicates that the influence of EBL on the carrier transport of micro-LED operating at low current density is much higher than that of the traditional high input/output LED. Table 1 also shows that with a relatively low Al composition in EBL, the electron barrier decreases with Al composition increased, which indicates that the downward of conduction band induced by polarization effect is larger than the increased band offset introduced by the higher Al composition. By further increasing the composition, the electron barrier increases along with composition, meaning that the band offset becomes dominant over the polarization effect. Compared with high current density, this turning point of composition is higher at low current density due to the enhanced polarization effect by less carrier screening. On the other hand, because of the original band offset between the last QB/*p*-GaN and the band bending, there are energy barriers for electron and holes exist at the interface even without the EBL. At 200 A/cm^2^, when the composition is lower than 0.20, the electron energy barrier is lower than the case of without EBL, but the hole barrier is higher approximately 64 meV. At 0.1 A/cm^2^, even with composition higher than 0.20, the electron barrier of AlGaN EBL (523 meV) is still 151 meV lower than the case of without EBL (674 meV), but the hole barrier is increased approximately 76 meV from 409 to 485 meV. These results indicate that both the electron confinement and hole injection could be deteriorated by the EBL with an incorrect composition, especially for micro-LED operating at low current density.

**Table 1:** Effective energy barrier height of different EBL structures for electrons and holes at 200 and 0.1 A/cm^2^, respectively

EBL	@ 200 A/cm^2^		@ 0.1 A/cm^2^	
	ΔE_electron_ (meV)	ΔE_hole_ (meV)	ΔE_electron_ (meV)	ΔE_hole_ (meV)
Al_0.20_Ga_0.80_ N	274	303	523	485
Al_0.15_Ga_0.85_ N	261	262	510	480
Al_0.10_Ga_0.90_ N	247	267	521	473
Al_0.04_Ga_0.96_ N	256	259	655	469
w/o EBL	265	239	674	409

For a deep analysis, band structures of micro-LEDs with Al_0.10_Ga_0.90_ N EBL and without EBL as representatives are illustrated in Fig. 8b, c. The EBL introduces two energy valleys at the interface of last QB/EBL and EBL/*p*-GaN for electron and hole accumulation, respectively, which can be confirmed by the carrier concentration diagram in Fig. 8d. Therefore, the electron confinement and hole injection are poor for this structure. When the EBL is removed, as shown in Fig. 8c, the energy barrier for electron is increased, and the energy valley for electron extracting and accumulation disappeares. These changes prevent electrons leakage more effectively, as confirmed in Fig. 8d. Meanwhile, the barrier height for hole injection is reduced, and the energy valley at the EBL/p-GaN interface is also removed. So, the hole can transport directly into the QW without facing large obstacle, as shown in Fig. 8c, d.

The above careful investigation suggests that without EBL may be a better structure for the micro-LED operating at low current density. Simulation results support our suggestion. Figure 8e illustrates the carrier current density at 0.1 A/cm^2^ with different EBL structures. When the Al composition of the EBL is reduced from 0.15 to 0.04, the total electron and hole current densities increase from 0.167 and 0.159 A/cm^2^ to 0.225 and 0.225 A/cm^2^, respectively. Moreover, when the EBL is completely removed, both the total electron and total hole current densities greatly increase to 0.528 A/cm^2^, which is approximately 3 times higher compared with the Al_0.15_Ga_0.85_ N EBL. This enhancement is contributed to the improved electron confinement and hole injection.

Figure 8f shows the IQE curves at low current density. When the Al composition of EBL is reduced from 0.15 to 0.04, the IQE values increase evidently due to the improved carrier transport. However, by removing the EBL, the IQE value experiences a slight decrease compared with Al_0.04_Ga_0.96_ N EBL. This can be explained by the carrier matching in two QWs. As indicated by the green arrows in Fig. 8e, a, perfect carrier matching occurred in both the two QWs with Al_0.04_Ga_0.96_ N EBL. With the increase of current density by removing EBL, the matching of electron and hole flux has been slightly broken in the first QW, where the electron current density is slightly higher than the hole. Therefore, the IQE is slightly reduced because of this carrier mis-matching in one QW.

The superiority of micro-LED without EBL is still remarkable due to the improved carrier transport. As shown in Fig. 8g, at 0.1 A/cm^2^, the integral EL intensities of micro-LED without EBL are 3.53, 3.23, and 2.38 times higher compared with the LED with Al_0.15_Ga_0.85_ N, Al_0.10_Ga_0.90_ N and Al_0.04_Ga_0.96_ N EBL, respectively. Moreover, as shown in the inset of Fig. 8g, the working voltages under 1 A/cm^2^ and 0.1 A/cm^2^ are reduced about 0.53 V and 0.57 V by removing the EBL, respectively. This improves the electrical efficiency, then finally increases the WPE of micro-LED. To further confirm that the EBL-free structure is a better design for micro-LED operating at low current density, another simulation is performed using the reported blue micro-LED structure with maximal known efficiency. The results and discussions can be found in the Supporting Materials (Additional file 1: Fig. S4a-d).

#### Optimized Structure for Micro-LED Operating at Low Current Density

**Fig. 9:**
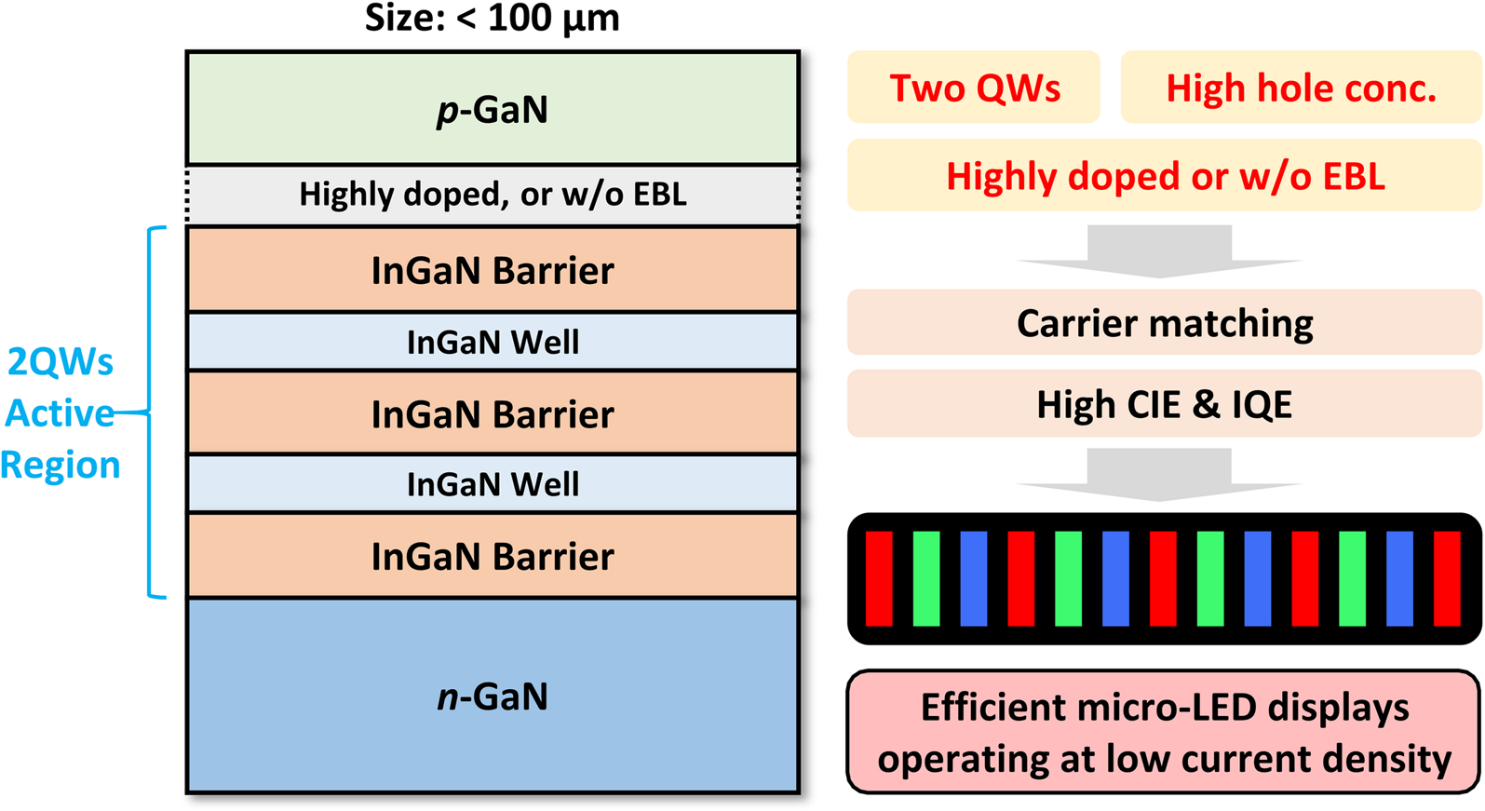
Schematic illustration of the optimized epitaxial structure designed specifically for the micro-LED emissive displays operating at low current density

Based on above simulation and analysis, the optimized epitaxial structure specifically designed for the efficient micro-LED emissive display operating at low current density is proposed, as shown in Fig. 9. Three principles must be followed. First, in contrast to the traditional large-size high-power nitride LED, the QW number of micro-LED should be reduced to just two, which has a better condition for the carrier matching, a more concentrated radiative emission, and higher IQE and WPE. Second, the *p*-type doing still needs to be enhanced due to the relatively low hole concentration and mobility compared with the electron in nitride, which demands a more efficient *p*-type doping strategy. Third, to improve the carrier transport and matching, the doping concentration of AlGaN EBL should be greatly enhanced, or the AlGaN EBL can be completely removed. Without using the AlGaN EBL, the electron confinement, hole injection, carrier matching, IQE, and WPE of the micro-LED can be greatly improved at low current density.

### Auger Recombination and SRH Non-radiative Recombination

Based on Eq. (3), except for radiative recombination, the SRH and Auger recombination also play critical roles in the IQE of LED. Hence, it is important to investigate the effects and mechanism regarding the SRH and Auger recombination for the micro-LED. In this part, the LED structure with 2QWs is still used, and all the simulation parameters are the same as mentioned in the Methods except for SRH lifetimes.

#### Negligible Auger and Prominent SRH Recombination

The SRH recombination rate can be expressed as follows [57]:

8 $$R_{{{\text{SRH}}}} = \frac{{np - n_{i}^{2} }}{{\tau_{p} \left( {n + n_{i} \exp \left( {\frac{{E_{T} }}{kT}} \right)} \right) + \tau_{n} \left( {p + n_{i} \exp \left( {\frac{{E_{T} }}{kT}} \right)} \right)}},$$

9 $$\tau_{p} = \frac{1}{{c_{p} N_{t} }}, \tau_{n} = \frac{1}{{c_{n} N_{t} }},$$

10 $$c_{p} = \sigma_{p} \nu_{p} , c_{n} = \sigma_{n} \nu_{n} ,$$

where *n*_*i*_ is the intrinsic carrier concentration, *τ*_*p*_ and *τ*_*n*_ are the hole and electron SRH lifetimes, respectively, *E*_*T*_ is the energy difference between the trap level and the intrinsic Fermi level, *c*_*p*_ and *c*_*n*_ are the capture coefficients for electron and hole, *N*_*t*_ is the trap density, *σ*_*p*_ and *σ*_*n*_ are capture cross sections for electron and hole, and *ν*_*p*_ and *ν*_*n*_ are the average thermal velocities of electron and hole, respectively. According to Eqs. (8)-(10), the SRH recombination of a trap is completely specified by its density, capture cross sections and energy level.

The Auger recombination rate is given by the following:

11 $$R_{{{\text{Auger}}}} = \left( {C_{n} n + C_{p} p} \right)\left( {np - n_{i}^{2} } \right),$$

where *C*_*n*_ and *C*_*p*_ are the Auger recombination coefficients.

Given that the injected hole and electron concentrations are much higher than the intrinsic carrier concentration in the undoped QWs (according to simulation result, the highest carrier concentration in QW is only approximately 10^7^ cm^−3^ in the absence of externally injected current), the SRH and Auger recombination rate can be further simplified as the following equations:

12 $$R_{{{\text{SRH}}}} = \frac{np}{{\tau_{p} n + \tau_{n} p}},$$

13 $$R_{{{\text{Auger}}}} = \left( {C_{n} n + C_{p} p} \right)np.$$

Equations (12) and (13) clearly show that *R*_SRH_ is in direct proportion to the first power of the carrier concentration, but *R*_Auger_ depends on the third power of the carrier concentration, that is, *R*_SRH_ is sensitive to low current density, while the *R*_Auger_ is more dominant at high current density.

**Fig. 10:**
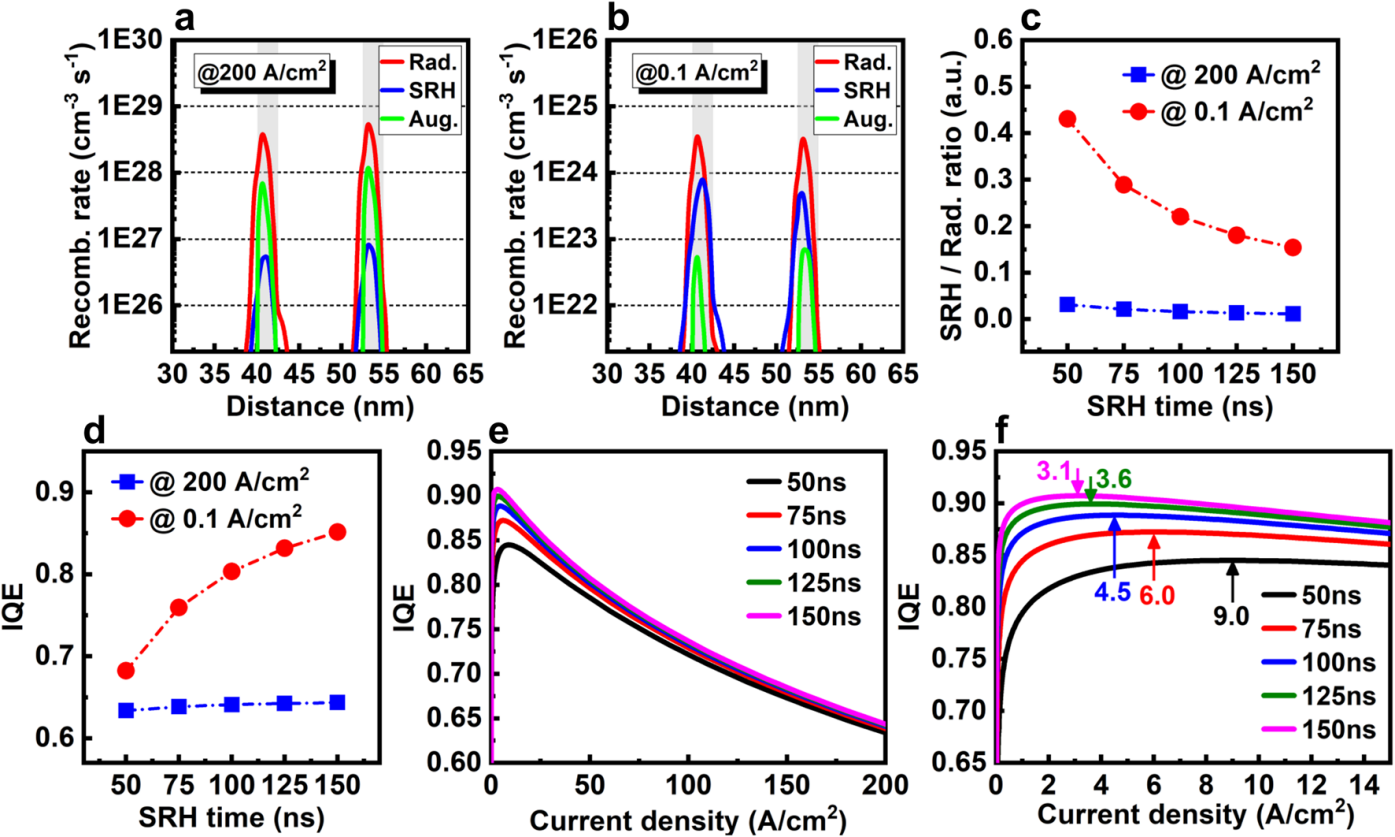
Radiative, SRH, and Auger recombination rates of 2QWs LED **a** at 200 A/cm^2^ and **b** at 0.1 A/cm^2^. **c** SRH/Radiative ratio with various SRH lifetimes at 200 and 0.1 A/cm^2^. **d** IQE values at 200 and 0.1 A/cm^2^, **e** IQE curves with large current density and **f** IQE curves with low current density at various SRH lifetimes

This theoretical analysis agrees with our simulation results. Figure 10a, b shows the calculated radiative, SRH, and Auger recombination rates at 200 and 0.1 A/cm^2^, respectively. At high current density, the Auger recombination rate (about 0.8–1.4 × 10^29^ cm^−3^ s^−1^) is comparable wiht the radiative rate (about 4.2–6.0 × 10^29^ cm^−3^ s^−1^). In fact, the substantial problem of efficiency droop at high drive currents is now widely acknowledged as caused by the Auger recombination [20]. While, at low current density, relatively, the Auger recombination rate dramatically decreases to two orders of magnitude lower (about 6.3–7.2 × 10^22^ cm^−3^ s^−1^) than the radiative recombination (about 3.7–4.0 × 10^24^ cm^−3^ s^−1^). Therefore, the Auger recombination should be negligible at low current density. Conversely, with the decrease of current density, the SRH recombination rate relatively increases from a small value at 200 A/cm^2^ (two orders of magnitude lower than radiative recombination) to a level comparable with the radiative emission at 0.1 A/cm^2^. As a result, the micro-LED operating at low current density requires improvement in the SRH or defect recombination instead of the Auger recombination.

#### Requirement for Low Defect Density

According to Eq. (9), SRH lifetimes, *τ*_*p*_ and *τ*_*n*_, are in inverse proportion to the density of defects *N*_*t*_. Therefore, the effect of defect density can be estimated by simply changing the SRH lifetime in the simulation. Figure 10c shows the calculated ratio of SRH/radiative recombination rate at various SRH lifetimes. With the decrease of SRH lifetimes from 150 to 50 ns, i.e., the increase of defect density, the SRH/radiative ratio slightly increases from 0.01 to 0.03 at 200 A/cm^2^, but greatly increases from 0.15 to 0.43 at 0.1 A/cm^2^. This means that a much larger percentage of carriers is consumed by the trapping defects at low current density. Therefore the efficiency is much more sensitive to the defect density at low current than high current density. The IQE results as shown in Fig. 10d, e confirm this trend. With the decrease of SRH lifetimes from 150 to 50 ns, IQE only decreases about 0.01 at 200 A/cm^2^, but dramatically decreases about 0.17 at 0.1 A/cm^2^. Moreover, as shown in Fig. 10f, with the decrease of SRH lifetime, the position of peak IQE also moves from 3.1 A/cm^2^ to a higher current density of 9.0 A/cm^2^, and the IQE curves become less steep and sharp, which means that the threshold/onset current is increased. This is disadvantageous for improving the efficiency of micro-LED at low current density.

Compared with the traditional large-size high-power LED working at high current density, the micro-LED operating at low current density is much more sensitive to defect density, and minimizing the defect recombination is of paramount importance for achieving a high efficiency. Therefore, the micro-LED requires a much more higher crystal quality of materials than the traditional LED, and poses large challenges for the epitaxial growth of the material and the fabrication of the device for the community.

## Conclusions

In summary, the operating behaviors, mechanisms and conditions of InGaN micro-LED operating at low current density are numerically investigated, and an optimized epitaxial structure specifically designed for the micro-LED display is proposed. Analysis of the polarization effect shows that micro-LED suffers a severer QCSE at low current density. Hence, improving the efficiency and controlling the emission color point are more difficult. Carrier transport and matching are analyzed to determine the operating conditions of micro-LED. It is shown that less QW number can improves the carreir matching and leads to higher efficiency and output power at low current density. Effectiveness of the EBL for micro-LED is analyzed, and electron confinement and hole injection are found to be improved simultaneously at low current density by removing the EBL. Moreover, simulaiton has shown that the Auger recombination is negligible, but the SRH recombination greatly influences the efficiency of micro-LED at low current density, which has raised higher requirements for the crystal quality of materials and the fabrication process of devices. Our numerical study can provide valuable guidance for creating efficient micro-LED display and promote future research in this area.

## Supplementary Information

**Additional file 1.** **Figure S1.** Space charge, electric field, transition energy and normalized EL spectra of micro-LED with 5QWs. **Figure S2.** EL spectra of InGaN-based red, green, and blue micro-LEDs at 0.1 and 200 A/cm^2^. **Table S1.** The 1931-CIE (x, y) color points created by combining the red, green, and blue micro-LEDs. ** Figure S3.** Carrier concentration and mobility of micro-LED with 5QWs. **Figure S4.** Simulation results from the micro-LED structure with maximal known efficiency.

## Data Availability

The data and the analysis in the current work are available from the corresponding authors on reasonable request.

## Abbreviations

Micro-LED: Micro-light-emitting-diode
QCSE: Quantum-confined Stark effect
EBL: Electron blocking layer
SRH: Shockley–Read–Hall
LCD: Liquid–crystal display
OLED: Organic light-emitting diode
TV: Televisions
VR: Virtual reality
AR: Augmented reality
QW: Quantum well
MQWs: Multiple quantum wells
EQE: External quantum efficiency
IQE: Internal quantum efficiency
QB: Quantum barrier
WPE: Wall-plug efficiency
CIE: Current injection efficiency
LEE: Light extraction efficiency
