# Supplementary material for: Designs of InGaN Micro-LED Structure for Improving Quantum Efficiency at Low Current Density
Source: Nanoscale Res Lett. 2021 Jun 3;16:99. doi: 10.1186/s11671-021-03557-4 (PMC8175512; doi:10.1186/s11671-021-03557-4)
Supplement: Supplementary file 1 — Additional file 1. Figure S1. Space charge, electric field, transition energy and normalized EL spectra of micro-LED with 5QWs. Figure S2. EL spectra of InGaN-based red, green, and blue micro-LEDs at 0.1 and 200 A/cm2. Table S1. The 1931-CIE (x, y) color points created by combining the red, green, and blue micro-LEDs. Figure S3. Carrier concentration and mobility of micro-LED with 5QWs. Figure S4. Simulation results from the micro-LED structure with maximal known efficiency. [file 11671_2021_3557_MOESM1_ESM.doc]

**Supporting Materials**

Designs of InGaN micro-LED structure for improving quantum efficiency at low current density

Shiqiang Lu^1^, Jinchai Li^1,2^,*, Kai Huang^1,2^, Guozhen Liu^1^, Yinghui Zhou^1^,

Duanjun Cai^1^,*, Rong Zhang^1,2^, and Junyong Kang^1,2^,*

*^1^ Fujian Key Laboratory of Semiconductor Materials and Applications, CI Center for OSED, College of Physical Science and Technology, Xiamen University, Xiamen 361005, China.*

*^2^ Future Display Institute of Xiamen, Xiamen 361005, China.*

* Corresponding authors:

jinchaili@xmu.edu.cn, dcai@xmu.edu.cn, and jykang@xmu.edu.cn


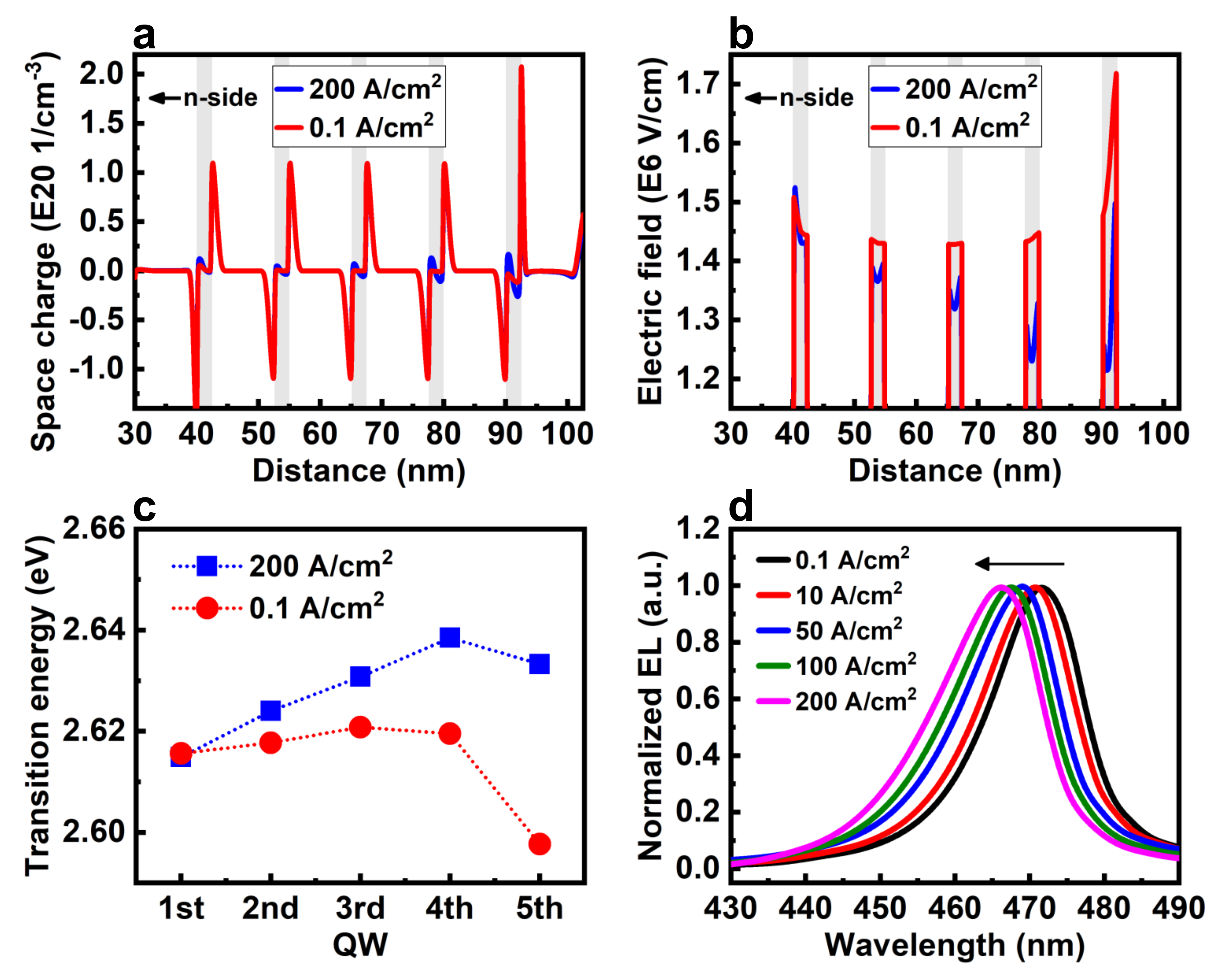


**Figure S1.** (a) Space charge distribution and (b) electric field

distribution of 5QWs at 200 and 0.1 A/cm^2^. (c) Transition energy of five QWs at 200 and 0.1 A/cm^2^. (d) Injection current density dependent normalized EL spectra of the micro-LED with 5QWs.

Figure S1a shows that the intrinsic polarization-induced space charges are mostly located at the interfaces of QWs. The positive and negative charges are located at the right- and left-side interfaces, respectively. As shown in Figure S1b, these charges lead to the strong internal polarization field that causes the QCSE. In contrast to the polarization-induced charges, the injected nonequilibrium holes and electrons assemble in the left- and right-side interfaces, respectively. Therefore, the nonequilibrium carriers could partially compensate the polarization-induced space charges and electrical field, then suppress the QCSE. Compared with the high current density, the population of nonequilibrium carriers is much lower at 0.1 A/cm^2^, thereby the screening effect of nonequilibrium carriers becomes weaker. Consequently, the electric filed in MQWs for 0.1 A/cm^2^ is larger than the case of 200 A/cm^2^. In a word, the QWs suffer a much stronger internal polarization field and a more serious QCSE at low current density than high current density. Due to this effect, the transition energies are lower at 0.1 A/cm^2^ than 200 A/cm^2^, leading to the observed shift of EL spectra, as shown in Figure S1c and d.


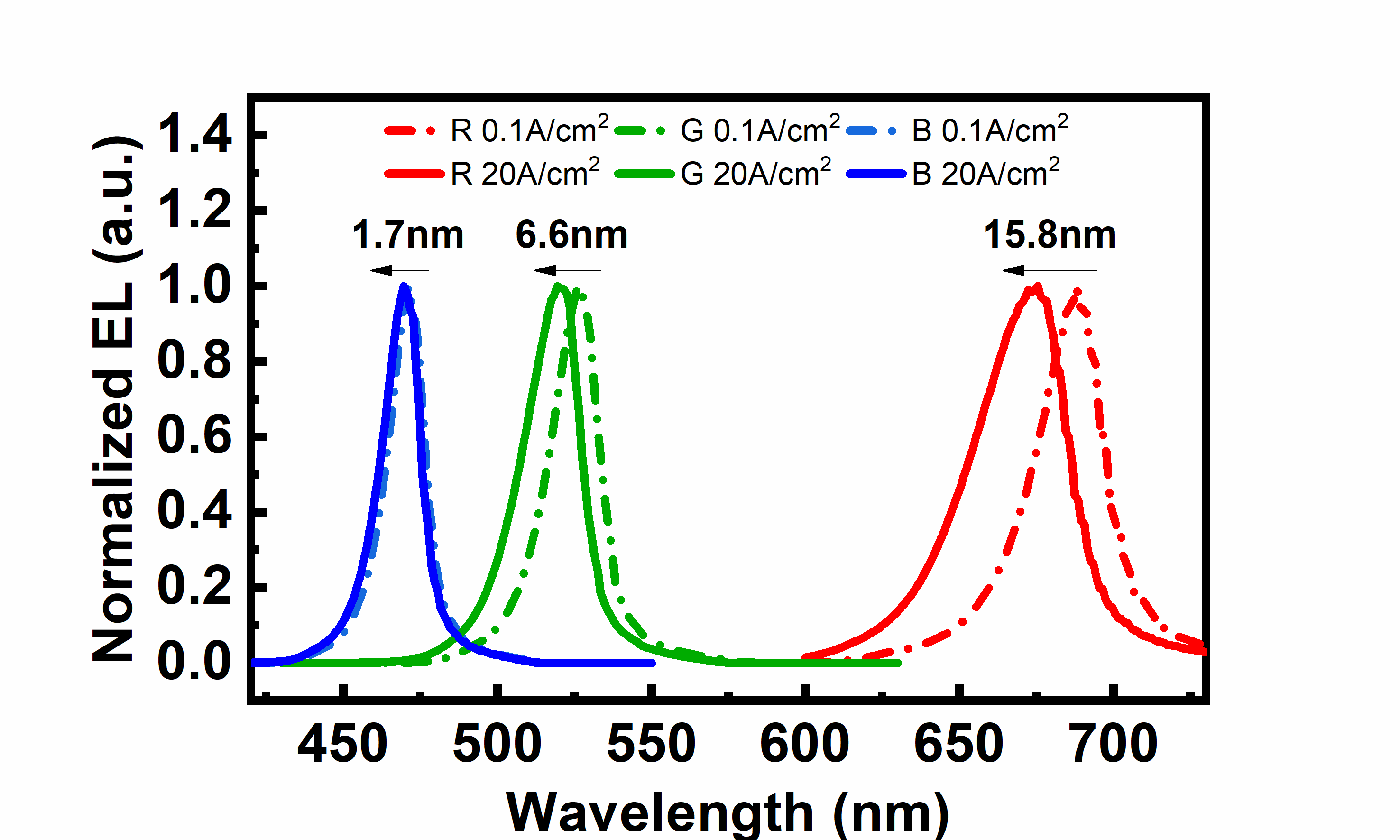


**Figure S2.** EL spectra of InGaN-based red, green, and blue micro-LEDs at 0.1 and 200 A/cm^2^. For green and red micro-LEDs, the In compositions of QWs are increased to 0.3 and 0.45, respectively.

**Table S1.** The 1931-CIE (x, y) color points created by combining the red, green, and blue micro-LEDs versus various current densities.

| **Current Density**  **(A/cm^2^)** | **CIE *x*** | **CIE *y*** |
| --- | --- | --- |
| **0.1** | 0.1908 | 0.3891 |
| **0.5** | 0.1746 | 0.3728 |
| **1** | 0.1714 | 0.3678 |
| **3** | 0.1676 | 0.3613 |
| **5** | 0.1727 | 0.3520 |
| **10** | 0.1851 | 0.3345 |
| **15** | 0.1973 | 0.3212 |
| **20** | 0.2084 | 0.3106 |


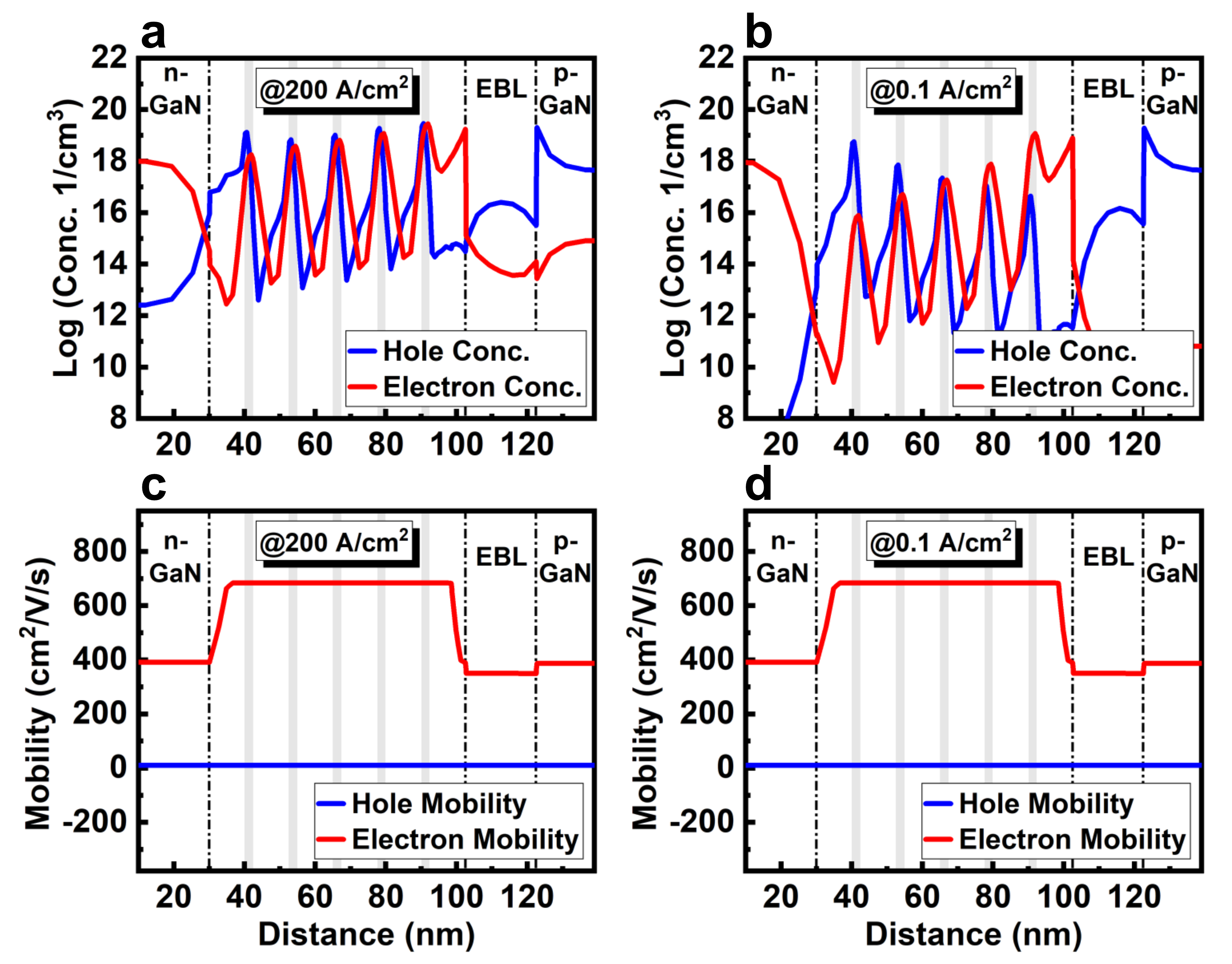


**Figure S3.** Carrier concentration distribution of LED with 5QWs (a) at 200 A/cm^2^ and (b) at 0.1 A/cm^2^. Carrier mobility distribution of LED with 5QWs (c) at 200 A/cm^2^ and (d) at 0.1 A/cm^2^.


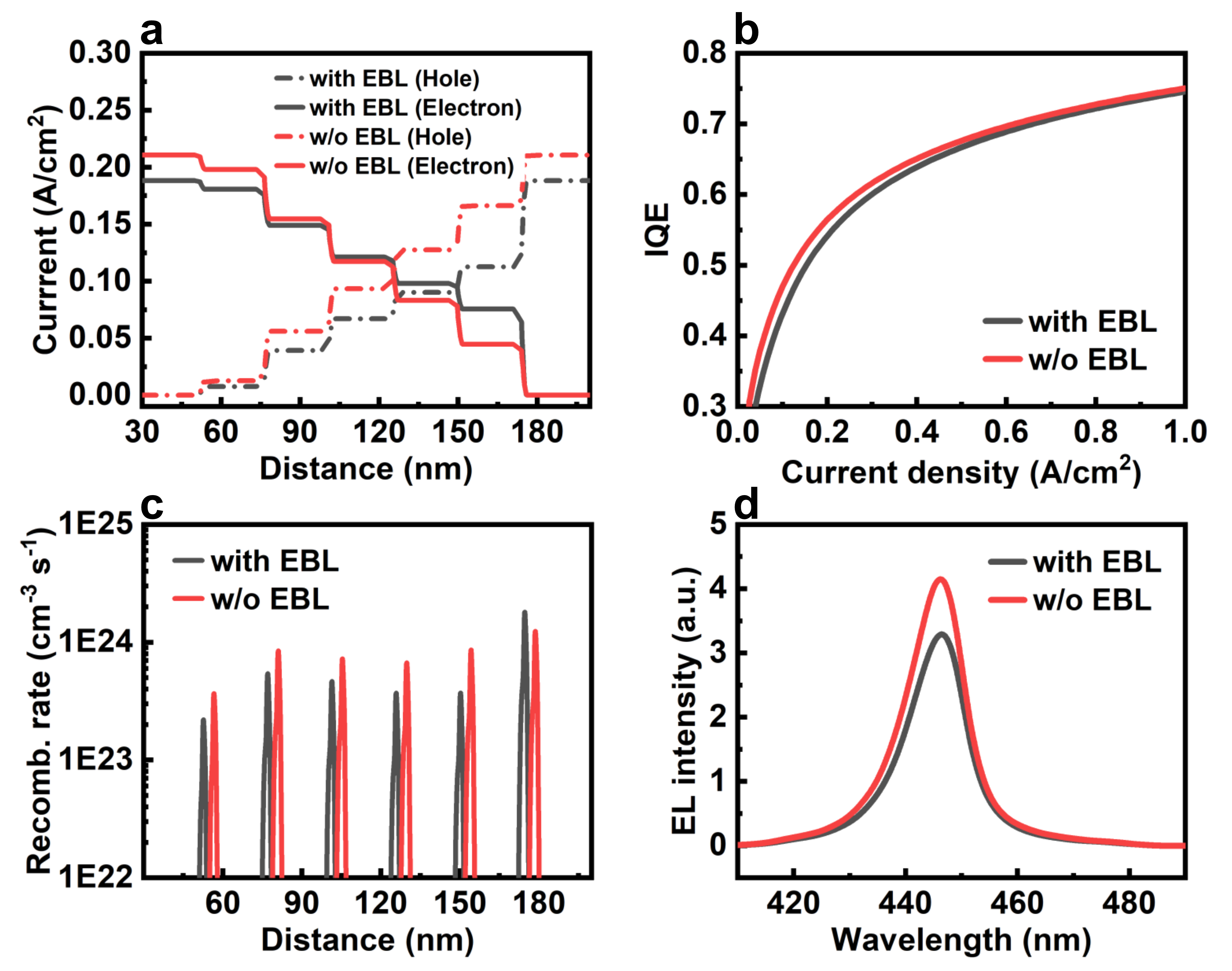


**Figure S4.** (a) The electron and hole current density at 0.1 A/cm^2^, (b) IQE curves at low current density, (c) radiative recombination rate at 0.1 A/cm^2^, and (d) EL spectra at 0.1 A/cm^2^ of the Ref-micro-LED with and without EBL.

In order to further confirm that the EBL-free structure is better at low current density, we have performed another simulation using the reported blue micro-LED structure which has maximal known efficiency to the best of our knowledge. The micro-LED structures used for these simulations are based on the works reported by Matthew S. Wong and his co-workers in University of California, Santa Barbara [*Applied Physics Express 10.3 (2017): 032101*] [*Optics express 26.16 (2018): 21324-21331*] [*Applied Physics Express 12.9 (2019): 097004*]. The micro-LED structure consisted of a 1.4 µm undoped GaN template layer, 4 µm of Si-doped n-GaN, and a 30-period Si-doped superlattice with 3 nm In_0.03_Ga_0.97_N and 3 nm GaN, which was then capped with 20 nm of GaN. The active region consisted of six MQWs with 2.4 nm InGaN wells and 22 nm GaN barriers with emission at 447 nm. Above the active region was a 26 nm Mg-doped AlGaN electron blocking layer, a 120 nm Mg-doped p-GaN layer, and a 17 nm Mg-doped p^+^-GaN contact layer. This structure was used as the reference micro-LED, and then the AlGaN EBL was removed as the new micro-LED to perform the comparison. As shown in Figure S4a-d, by removing the EBL, both the electron and hole current density are increased from 0.188 to 0.211 A/cm^2^ for the micro-LED at 0.1 A/cm^2^. And the IQE value of micro-LED was slightly improved without EBL at low current density. More importantly, the integral intensity of radiative recombination rate and EL intensity are increased about 24.6% and 24.5% at 0.1 A/cm^2^ by removing the EBL structure. Though these improvements are lower compared with the results in main manuscript, still, they can prove that the micro-LED structure without EBL is more suitable for operating at low current density.
